# Lactoferrin treatment activates acetylcholinesterase, decreasing acetylcholine levels in non‐small cell lung cancer (NSCLC) cell culture supernatants, inhibiting cell survival

**DOI:** 10.1002/2211-5463.70125

**Published:** 2025-09-23

**Authors:** Stuti Goel, Caroline Wozniak, Aya Sabri, Ben Haddad, Brooke Lopo, Alvaro Cobos, Sarah Sarofim, Jeffrey Guthrie, Deborah Heyl, Hedeel Guy Evans

**Affiliations:** ^1^ Chemistry Department Eastern Michigan University Ypsilanti MI USA

**Keywords:** acetylcholine, cell viability, lactoferrin, lung cancer, p53, vascular endothelial growth factor

## Abstract

Lactoferrin (Lf) is a multifunctional glycoprotein of the transferrin family which has shown to efficiently block cell migration and/or invasion in a wide range of cancer cell models. The objective of this study was to further understand how Lf targets cancer cells by examining the effect of acetylcholine (ACh) levels on Lf signaling using A549 (p53 wild‐type) and H1299 (p53‐null) nonsmall cell lung cancer (NSCLC) cell lines. Treatment with Lf reduced cell viability more effectively in A549 cells than in H1299 cells. The half maximal inhibitory concentration (IC_50_) of Lf for A549 and H1299 was 8.97 ± 1.4 and 35.03 ± 4.2 mg·mL^−1^, respectively. To uncover the potential molecular mechanism involved in the decreased cell viability observed in A549 cell following Lf treatment, the activity of tumor suppressor (p53), acetylcholinesterase (AChE), and ACh levels were measured. Treatment of A549 cells with Lf led to ~ 1.50‐fold activation of p53, ~ 1.60‐fold activation of AChE, and ~ 1.80‐fold decrease in ACh levels. Vascular endothelial growth factor (VEGF) levels also decreased in cell culture supernatants upon treatment with Lf in both cell lines, and in A549 cells, the decrease occurred in a manner dependent on p53 and AChE. Given previous reports on the role of Lf in apoptosis induction, we examined AKT activity following Lf treatment and showed that AKT activity decreased ~ 1.95‐fold in A549 cells and ~ 1.50‐fold in H1299 cells. Furthermore, Lf‐induced activation of caspase‐3 was diminished by A549 cell cotreatment with siRNA targeted against p53 and/or AChE and increased by inhibiting the function of VEGF and/or AKT in both cell lines. In conclusion, this study identifies a mechanism wherein ACh concentrations in the cell culture supernatant attenuate the impact of Lf on NSCLC cell viability. These findings provide preliminary insight into the complex actions of Lf and suggest that the Lf‐AChE‐ACh pathway may warrant further study as a potential target in NSCLC.

## Graphical abstract

Representation of the suggested mode of action of lactoferrin (Lf) in nonsmall cell lung cancer (NSCLC) A549 cells. Lf induces activation of caspase‐3 by activating p53 and AChE leading to decreased ACh concentrations. In turn, ACh signaling leads to activation of VEGF and AKT and blocking of caspase‐3. Thus, Lf action ultimately leads to increased apoptosis and decreased cell survival.

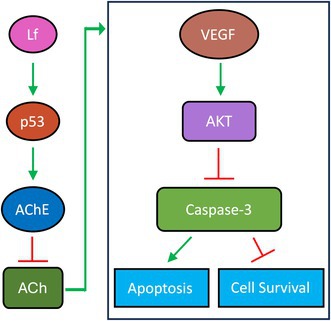

**Abbreviations**
ACh: acetylcholine
AChE: acetylcholinesterase
Lf: lactoferrin
VEGF: vascular endothelial growth factor

Worldwide, cancer is a major medical challenge and a growing public health issue [1, 2]. The most frequently diagnosed malignant tumor and a leading cause of cancer deaths is lung cancer that can be divided into nonsmall cell lung cancer (NSCLC) and small cell lung cancer (SCLC) [2]. NSCLC, categorized into squamous cell carcinoma, adenocarcinoma, and large cell carcinoma, accounts for ~ 85% of all lung cancer cases [1, 3].

Lactoferrin (Lf) is a member of the transferrin family that is capable of binding and transferring iron ions [4]. Lf is a natural 80 kDa cationic glycoprotein with anticancer properties [5]. Due to its cationic properties, Lf is thought to form electrostatic interactions with negatively charged cell surface receptors promoting cancer cytotoxicity [5, 6]. Lf was shown to exhibit high selectivity towards inhibiting the growth of breast cancer cell lines but not normal breast cells [6] and anticancer activity against human lung adenocarcinoma with no effect on normal human bronchial epithelial cells [7]. Using a model of oral squamous cell carcinoma, bovine Lf (bLf) was reported to increase the levels of phospho‐p53 inducing cell cycle arrest [8].

In apoptotic MCF‐7 cells treated with cisplatin, acetylcholinesterase (AChE) was shown to be a downstream component of p53 [9, 10]. Expression of AChE was upregulated in response to activation of p53 and abolished by blocking p53 expression using siRNA [9]. The classical key role of AChE is known to be the catalytic hydrolysis of cholinergic neurotransmitters; however, more recently, nonclassical functions of the enzyme have emerged suggesting that AChE might be a potentially promising regulator of apoptosis [10] and anticancer therapeutic [11, 12]. Low levels of AChE in some tumor cells that are not sensitive to apoptosis were reported to protect cells against apoptosis [10, 11, 12]. Previously, we reported that insulin‐like growth factor binding protein‐3 (IGFBP‐3) binds hyaluronan and blocks its interaction with the main cell surface receptor for hyaluronan, CD44, resulting in increased AChE expression and activity in a p53‐dependent manner leading to apoptosis and decreased NSCLC cell survival [13]. Additional studies on the noncanonical role of AChE in cancer biology have been recently reported. For example, a bioinformatics analysis examined the pan‐cancer expression signature of AChE using a data‐driven approach and found significant dysregulation of AChE in a number of cancer tissues compared to normal [14]. In lung adenocarcinoma, cholinergic metabolic activation was suggested to be a hallmark of brain metastasis, and inhibition of AChE with donepezil markedly reduced brain metastasis [15]. Moreover, another study found that AChE content and composition differed in Jurkat cells versus normal T lymphocytes providing opportunities for investigating new therapeutic approaches for T‐cell leukemia [16].

In examining A549 (p53 wild‐type) and H1299 (p53‐null) NSCLC cell lines used in this study, we showed that the levels of AChE were minimal in H1299 cell media as compared to the media of A549 cells [13]. We subsequently reported that the levels of acetylcholine (ACh) were higher in the media of H1299 cells than in A549 cell media and that knockdown of AChE led to increased ACh levels in the media of A549 but not of H1299 cells [17]. These results were consistent with previous reports showing that ACh can be secreted by lung cancer cells into the extracellular environment, increasing cancer cell growth in lung tumors [18, 19]. ACh functions as an autocrine growth factor by binding to nicotinic and muscarinic receptors on lung cancer cells, accelerating their proliferation [20]. ACh was reported to have mitogenic effects in A549 and in H1299 NSCLC cells, upregulating the levels of matrix metalloproteinases and decreasing the levels of E‐cadherin in A549 human NSCLC [10, 20].

Previously, we reported that vascular endothelial growth factor (VEGF) can be upregulated via the α7‐nicotinic acetylcholine receptor (α7nAChR) and/or β‐adrenergic‐receptors (β‐ARs) and downregulated by p53 in response to nicotine treatment of NSCLC cells [21]. We also showed that decreased p53 activity, and conversely, increased VEGF levels and activities of PI3K, AKT, NFκB, led to increased NSCLC cell survival and decreased apoptosis [21, 22, 23]. These results are consistent with the role of the PI3K/AKT pathway as a cancer‐inducing signaling cascade that increases cell proliferation and resistance to apoptosis [20, 24, 25, 26, 27]. We also reported a mechanism by which nAChRs and β‐ARs can lead to regulation of PI3K/AKT signaling and chemoresistance in NSCLC cells [22]. More recently, we showed that increased matrix metalloproteinase‐9 (MMP9) levels in A549 and H1299 cells enhanced PI3K and ERK1/2 signaling, decreased p53 activity, leading to decreased apoptosis and increased cell survival [28].

Lf is a natural iron‐binding protein that is generally considered safe with minimal adverse effects with the potential as an alternative pharmaceutical molecule to chemotherapeutic agents [5]. Lf shows promise in cancer prevention and treatment due, in part, to its ability to trigger apoptosis in cancer cells [4, 29, 30, 31]. Its ability to target cancer cells selectively by efficiently blocking migration and/or invasion in a wide range of cancer cell models is a significant advantage [5, 6, 8, 29, 31, 32]. The mechanisms by which Lf targets cancer cells are currently under active investigation. Results from this work are significant because they provide new insights into a mechanism involving activation of AChE and inhibiting VEGF function in the regulation of Lf signaling in NSCLC cells. Our findings also show that Lf treatment of NSCLC cells led to activation of AChE and decreased ACh levels, inhibiting cell viability and increasing apoptosis, possibly pointing to the Lf‐AChE‐ACh pathway as a potential therapeutic target. This work might contribute to the present therapeutic status of this cancer, provide valuable information on the potential clinical applications of Lf in NSCLC therapy, offer potential new avenues for treatment and enhancing existing therapies, and may help in the development of anticancer drugs with greater drug efficacy.

The hypothesis of this study is that Lf induces activation of caspase‐3 in NSCLC A549 cells by activating p53 and AChE leading to decreased ACh concentrations in the cell culture supernatant. To test this hypothesis, we chose the A549 and H1299 cell lines since A549 cells are known to be p53 wild‐type and H1299 cells are known to be p53‐null [33]. Moreover, we have previously reported that the levels of AChE are minimal in H1299 cell media as compared to the media of A549 cells [13], that the levels of ACh are higher in the media of H1299 cells than in A549 cell media, and that knockdown of AChE led to increased ACh levels in the media of A549 cells but not in H1299 cells [17].

## Materials and methods

### Materials

Most of the material used in this study was purchased as we reported earlier [13, 34, 35, 36, 37, 38]. Phosphate‐buffered saline (PBS), nitrocellulose membranes, human Lf (L1294), MISSION human ACHE (esiRNA1, EHU072891), and AKT inhibitor (Calbiochem, San Diego, CA, USA) were purchased from Sigma‐Aldrich, St. Louis, MO, USA. The caspase‐3 (cleaved) colorimetric in‐cell ELISA kit (62218), Halt protease and phosphatase inhibitor cocktail, BCA protein assay kit, super signal west pico luminol (chemiluminescence) reagent, α‐tubulin mouse monoclonal antibody (DM1A), goat anti‐mouse IgG (H + L) superclonal secondary antibody, HRP conjugate (A28177), 3,3′,5,5′‐tetramethylbenzidine (TMB), lipofectamine 2000 transfection reagent, human IgG (hIgG) isotype control, and goat anti‐rabbit IgG (H + L) secondary antibody (HRP, 31466) were from Thermo Fisher, Waltham, MA, USA. SignalSilence p53 siRNA I (6231), SignalSilence control siRNA (Unconjugated, 6568), and p53 rabbit antibody (9282) were purchased from Cell Signaling Technology, Danvers, MA, USA. Goat anti‐AChE antibody (ab31276), rabbit anti‐goat IgG H&L (HRP) (ab6741), and donkey anti‐mouse IgG (HRP) (ab205724) were purchased from Abcam, Cambridge, MA, USA. Human/primate VEGF antibody (MAB293‐100) was purchased from R&D Systems, Minneapolis, MN, USA.

### Cell culture

Human NSCLC cell lines, A549 (ATCC CCL‐185) and H1299 (ATCC CRL‐5803), were obtained from the American Type Culture Collection (ATCC, Manassas, VA, USA). Cells were cultured as we reported previously [13, 34, 35, 36, 39, 40] in DMEM/F12 media in the presence of 10% fetal bovine serum (FBS), 50 U·mL^−1^ penicillin, and 50 U·mL^−1^ streptomycin at 37 °C, 95% humidity, and 5% CO_2_. The cells were counted using a hemocytometer following trypan blue staining. A549 and H1299 cells were passaged at 80–90% confluence by standard trypsinization at ~ 1 : 5–1 : 10 dilutions and cultured for up to 3 weeks. Following thawing, the passage number remained at or below 20. Cells were regularly checked for morphological changes and p53 status using ELISA and western blotting. In certain experiments, cells were cultured for 24, 48, and 72 h. The 72‐h incubation period is needed for cell culture experiments studying the cells' supernatant (secretomes) since a longer incubation period allows for a greater accumulation and better detection of secreted factors in the conditioned media.

### MTT assay

The MTT reduction assay (Sigma‐Aldrich) was used to measure cell viability in 96‐well plates as we previously reported [17, 22, 28, 37, 38]. Viable cells are able to convert MTT into purple formazan crystals. Untreated cells were used as a positive control while wells containing only DMSO and cell‐free culture media were used as negative controls. After incubation, the formazan crystals were dissolved and the absorbance was measured at 570 nm in a plate reader and normalized to cell number (absorbance/cell number). All absorbance measurements were in the linear range. Statistical analysis was carried out with graphpad prism version 10.5.0 for Windows.

### Caspase‐3 assay

For the caspase‐3 (cleaved) colorimetric assay, activated (cleaved) caspase‐3 and α‐tubulin were measured simultaneously in whole cells in triplicate using an in‐cell ELISA assay (Thermo Fisher), as we reported previously [41, 42]. The assay enables simultaneous measurement of target and normalization proteins directly in fixed cells. Briefly, A549 and H1299 cells were seeded in 96‐well plates, treated under the indicated experimental conditions, and fixed without removing the cells. Wells were incubated with a primary antibody specific for cleaved (activated) caspase‐3, followed by an HRP‐conjugated secondary antibody and colorimetric substrate, with absorbance measured at 450 nm. In the same wells, α‐tubulin was detected using a second antibody pair to provide an internal normalization control for cell number and protein concentration. Caspase‐3 activity was expressed as the absorbance ratio of cleaved caspase‐3 to α‐tubulin and normalized to untreated controls for comparison across treatments.

### Quantitation of ACh concentrations

The choline/acetylcholine assay kit (ab65345) was used to measure the concentration of ACh according to the manufacturer's recommendation and as we reported previously [17, 43, 44]. The amount of ACh in the samples was calculated by subtracting choline from total choline (choline + ACh). Briefly, culture supernatants from A549 and H1299 cells were collected at the indicated time points and kept on ice. Media samples were divided into two aliquots: one treated with AChE to convert ACh into choline, and one untreated to measure baseline choline. Both aliquots were incubated with the supplied enzyme and reaction mixes, resulting in a colorimetric product quantified at 570 nm using a microplate reader. ACh concentrations were calculated by subtracting the choline‐only signal from the total signal obtained after AChE treatment, using a standard curve generated from known ACh concentrations. Data were normalized to protein concentrations. Negative controls included using assay buffer alone while ACh standards served as a positive control.

### AChE activity

The activity of AChE in the conditioned media was measured using the AChE activity assay kit (MAK119) according to our previous methods [13, 17, 43, 44] and those previously reported [20, 45]. The colorimetric product was proportional to the activity of AChE in the samples. Briefly, cell culture supernatants from A549 and H1299 cells were collected at the indicated treatments and time points and assayed immediately or stored at −80 °C. For each sample, aliquots of media were incubated in a 96‐well plate with the provided reaction mix containing ACh as a substrate and Ellman's reagent [5,5′‐dithiobis(2‐nitrobenzoic acid), DTNB], which reacts with the thiocholine released during substrate hydrolysis to produce a yellow chromophore. Absorbance was measured at 412 nm, and the rate of increase in absorbance was used to calculate AChE activity based on a standard curve generated with known amounts of AChE. Activity values were normalized to viable cell number from parallel wells to account for differences in cell density and viability. Negative controls included using assay buffer alone while AChE served as a positive control.

### P53 transcription factor activity assay

The p53 activity was assayed using the human p53 transcription factor activity assay kit (RayBio, Peachtree Corners, GA, USA; TFEH‐p53) as we previously reported [23, 28, 37, 38]. Briefly, double‐stranded oligonucleotides containing the p53 binding sequence were bound to 96‐well plates to capture the active p53 in whole cell lysates. Briefly, lysates were prepared from A549 and H1299 cells harvested under the indicated conditions. Equal amounts of protein were incubated in 96‐well plates precoated with a double‐stranded DNA consensus sequence specific for the p53 binding site. Activated p53 present in the extracts bound to the immobilized oligonucleotides, and the bound complex was detected with a primary antibody specific for p53 followed by HRP‐conjugated secondary antibody. Colorimetric detection was performed with a TMB substrate, and absorbance was read at 450 nm using a microplate reader. P53 activity was expressed as relative absorbance units normalized to protein content, with A549 extracts serving as a positive control and H1299 extracts (p53‐null) serving as a negative control.

### Quantitation of protein levels and normalization to α‐tubulin

The Human p53 ELISA Kit (ab171571) was used to quantitate total p53 levels. Briefly, A549 and H1299 cell lysates were prepared in the supplied lysis buffer, clarified by centrifugation, and equal amounts of total protein were added to 96‐well plates precoated with an anti‐p53 capture antibody. Bound p53 was detected with a biotinylated detection antibody and HRP‐streptavidin conjugate, followed by TMB substrate development. Absorbance was measured at 450 nm, and p53 concentrations were calculated from a standard curve generated using recombinant p53 protein, with results normalized to the total protein concentration. The human acetylcholinesterase/ACHE DuoSet ELISA (DY7574‐05) was used to quantitate AChE. Briefly, 96‐well plates were coated with the capture antibody specific for human AChE, blocked to prevent nonspecific binding, and incubated with conditioned media or cell lysates collected from A549 and H1299 cells. After washing, wells were treated with a biotinylated detection antibody, followed by streptavidin‐HRP and TMB substrate. Absorbance was measured at 450 nm with wavelength correction at 540 nm, and AChE concentrations were determined from a recombinant AChE standard curve. Results were normalized to the total protein concentration in the wells. The human α‐tubulin ELISA kit (A312476) was used to quantitate α‐tubulin. Cell lysates were added to wells precoated with an α‐tubulin capture antibody, and bound protein was detected with an HRP‐conjugated secondary antibody and TMB substrate. Absorbance was read at 450 nm, and concentrations were calculated from a recombinant α‐tubulin standard curve. Once the concentrations of p53, AChE, and α‐tubulin in each sample were quantitated using their respective ELISA standard curves, the ratios of total p53 or AChE to α‐tubulin were then calculated and plotted for each sample.

### VEGF concentration determination

The concentration of VEGF in the cell culture media was measured as we previously reported [21] using the human VEGF solid‐phase sandwich ELISA kit (Thermo Fisher, KHG0111). The media from A549 and H1299 cells was added to 96‐well plates precoated with a VEGF‐specific capture antibody. After incubation and washing, a second biotinylated anti‐VEGF detection antibody was added, followed by streptavidin‐HRP and the TMB substrate. Absorbance was measured after addition of streptavidin‐HRP and the substrate solution at 450 nm. VEGF concentrations were determined from a standard curve generated with recombinant human VEGF. Results were normalized to total protein concentration. The intensity of the signal was directly proportional to the concentration of VEGF in the media. Negative controls included using assay buffer alone while recombinant human VEGF served as a positive control.

### AKT assay

The AKT kinase activity ELISA kit (Abcam) was used to quantitate the AKT activity according to the manufacturer's instructions as we reported earlier [17, 22, 23, 37, 38, 40]. The assay uses a synthetic peptide as a specific AKT substrate and a polyclonal antibody that targets the phosphorylated substrate. Briefly, A549 and H1299 cells were lysed in the supplied kinase extraction buffer, and equal amounts of protein were incubated in 96‐well plates coated with a specific AKT substrate peptide. Active AKT in the samples phosphorylated the immobilized substrate, which was then detected with a phospho‐AKT substrate‐specific antibody followed by an HRP‐conjugated secondary antibody. Colorimetric detection was performed using TMB substrate, and absorbance was read at 450 nm. AKT kinase activity was calculated from a standard curve generated with recombinant active AKT and normalized to the total protein concentration. Negative controls included using assay buffer alone while recombinant AKT served as a positive control.

### Dot blotting

Total protein samples of the conditioned media, 3 μL of 600 μg·mL^−1^, obtained after the indicated cell treatments, were spotted directly onto a nitrocellulose membrane and allowed to air‐dry as we reported earlier [13, 34, 36, 40, 44]. Briefly, the membrane was blocked in 5% nonfat milk in TBS‐Tween (TBST) for 1 h at room temperature (RT) then incubated with goat anti‐AChE antibodies overnight at RT. The membrane was washed then incubated with anti‐goat IgG‐HRP for 30 min at RT. After washing, the amount of AChE on the membrane was detected using SuperSignal West Pico luminol (chemiluminescence) reagent. The blots were imaged with a Bio‐Rad (Hercules, CA, USA) molecular imager. Recombinant human AChE was used as a positive control, and distilled water was used as a negative control.

### Western blotting

Cell lysates were collected as indicated and analyzed according to our earlier methods [13, 17, 22, 23, 34, 37, 38, 40]. Briefly, samples were separated by 12% SDS/PAGE, then transferred onto a nitrocellulose membrane. Following blocking with 5% nonfat milk in TBST and washing steps, the membrane was incubated with primary antibodies targeting p53, AChE, or α‐tubulin overnight at 4 °C. The membrane was then washed and incubated with the corresponding HRP‐conjugated secondary antibodies diluted according to the instructions provided by the manufacturers for 1 h at RT. After washing, the blots were next developed using super signal west pico luminol (chemiluminescence) reagent and imaged using a Bio‐Rad molecular imager. The same blot was stripped and reprobed using Restore western blot Stripping Buffer (Thermo Fisher) according to the instructions provided by the manufacturer. The signals from p53 and AChE were normalized to α‐tubulin.

### siRNA transfection

Transfections were carried out according to our earlier methods [13, 17, 23, 28, 37, 40]. Control siRNA, p53 siRNA, or AChE siRNA (100 nm) were each mixed with Lipofectamine 2000 transfection reagent diluted in Opti‐MEM Reduced Serum Media (Thermo Fisher) and incubated at RT for 20 min to allow complex formation. The mixtures were then added to the cells that were then incubated for 12 h at 37 °C, followed by the specific indicated treatments. Each measurement represents the mean ± SD of three‐five independent experiments, each performed at least in triplicate. Knockdown efficiency was confirmed by ELISAs and/or western blotting.

### Statistical analysis

The analysis was performed as we previously reported [13, 17, 22, 23, 35, 36, 37, 38, 40]. To evaluate the statistical differences, the Mann–Whitney or an ordinary one‐way ANOVA followed by Tukey's *post hoc* multiple comparison test was performed. For bar graph analyses, the Mann–Whitney test was used to compare differences between two individual bars, whereas an ordinary one‐way ANOVA followed by Tukey's *post hoc* multiple comparison test was performed to evaluate differences across three or more bars within a dataset. All the statistical tests were two‐sided, and a *P* value of < 0.05 was considered statistically significant in all cases. graphpad prism (GraphPad Software, San Diego, CA, USA; 10.5.0, Prism) was used for the statistical analysis.

## Results and Discussion

### Lf treatment of A549 cells decreased cell viability more effectively than treatment using H1299 cells

**Fig. 1:**
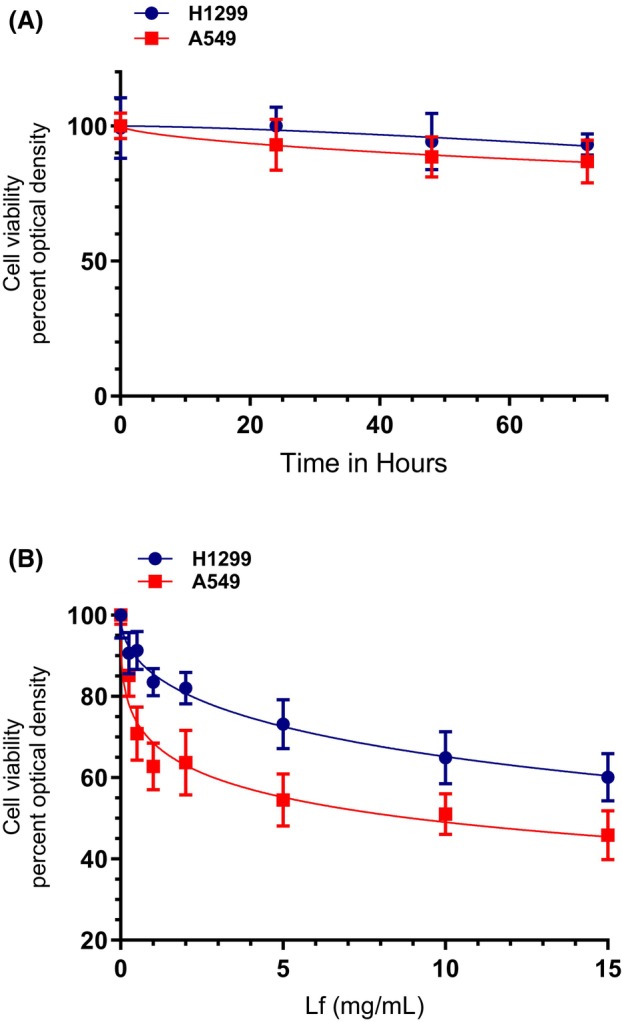
Treatment with lactoferrin (Lf) was more effective at decreasing A549 cell viability compared to H1299. Cells were grown to confluence then serum‐starved overnight. The cell monolayers were then incubated in serum‐free media for 72 h in the absence of any treatment (A) and in the presence of increasing concentrations of Lf for 72 h (B). Cell viability (A, B) was then measured and normalized to cell number (absorbance/cell number), as described in the Materials and methods section. Optical densities (A, 570 nm) were normalized for the curves by expressing each point relative to the best fitted *E*_max_ value (set to 100%) or by expressing each point relative to control samples without Lf (B, set to 100%). The data were then plotted as a function of time (A) or increasing Lf concentrations (B) and fit using a nonlinear regression curve fitting approach. Data were processed using the graphpad prism 10.5.0 software and presented as the mean ± SD of three independent assays, each carried out in triplicate.

The anticancer and antimetastatic activities of Lf against a variety of human cancers, both *in vitro* and *in vivo*, suggest that Lf may be considered a promising anticancer agent [5, 6, 7, 8, 29, 46]. To be consistent with previously reported conditions in the literature using A549 cells [47], A549 and H1299 cells were cultured in DMEM/F12 media in the presence of 10% FBS in 96‐well plates. After the cells reached 80% confluence in the wells, the media was aspirated then 100 μL of new media (FBS‐free) without or with 0–15 mg·mL^−1^ Lf was added to the wells. The cells were then incubated for 72 h, and the cell viability was measured as described in the Materials and methods section (Fig. 1, Table 1).

**Table 1:** Cell viability summary.

Condition	A549 cells	H1299 cells
Baseline (72 h, untreated)	~ 13% reduction	~ 7% reduction
Lf treatment (15 mg·mL^−1^, 72 h)	~ 55% inhibition	~ 40% inhibition
IC_50_ (mg·mL^−1^)	8.97 ± 1.4	35.03 ± 4.2

Cell viability of the two cell lines was not significantly affected under these conditions over a period of 72 h (Fig. 1A). Cell viability was reduced ~ 7% for H1299 cells and ~ 13% for A549 cells over a period of 72 h (Fig. 1A). A549 cell treatment with 15 mg·mL^−1^ Lf resulted in ~ 55% inhibition of cell viability while the same treatment reduced H1299 cell viability ~ 40% (Fig. 1B). The half maximal inhibitory concentration (IC_50_) of Lf for A549 and H1299 was calculated to be 8.97 ± 1.4 and 35.03 ± 4.2 mg·mL^−1^, respectively (Fig. 1B). These results are consistent with a previous report showing that recombinant human Lf inhibited A549 cell growth and induced apoptosis by activation of caspase‐3 [29]. To our knowledge, this is the first report comparing cell viability of A549 and H1299 under our conditions as a function of increasing Lf concentrations.

### Treatment of A549 cells with Lf led to activation of p53 and AChE and decreased ACh levels

Multiple mechanisms have been reported for Lf to promote its antitumor effects [4, 5, 6, 7, 8, 29, 48]. Among these mechanisms is the upregulation of a key tumor suppressor protein, p53, activation of caspase‐3, and apoptosis induction [48]. Previously, we found that hyaluronan‐CD44 signaling can be blocked by IGFBP‐3 via a mechanism that depends on p53 and AChE, and that treatment of A549 cells transfected with p53 siRNA with IGFBP‐3 led to diminished levels and activity of AChE in the media [13]. In addition, we found that the levels of AChE were lower in the media of H1299 cells (p53‐null) as compared to A549 cell media [13, 17]. More recently, we showed that treatment of A549 cells with leptin increased ACh levels and inhibited the activities of p53 and AChE [44]. AChE may function as a tumor suppressor in part due to the catalytic hydrolysis of ACh [10, 49, 50, 51]. The activity of AChE was reported to be decreased in lung cancer, likely leading to increased ACh levels and lung cancer growth [10, 49, 50, 51]. Using antisense oligonucleotides to block expression of AChE or pharmacological inhibition of AChE was shown to prevent apoptosis [10, 52].

To examine a potential mechanism through which Lf decreases cell viability more effectively in A549 cells as compared to H1299 cells (Fig. 1), cells were grown in FBS‐supplemented media for 24 h, then incubated in serum‐free media overnight. The media was then replaced with fresh serum‐free media, and the cells were treated with Lf (10 mg·mL^−1^) as indicated for 72 h. This Lf concentration was chosen to be consistent with previous reports using A549 cells to show that Lf at a concentration of 10 mg·mL^−1^ promoted increased miR‐146a expression in A549 cells and mouse lung tissue [47]. Lf was also shown to exert cytotoxic effects on human liver cancer cells [30], and at a concentration of 10 mg·mL^−1^, the proliferation of HepG2 and Hep3B cells was completely inhibited while no significant effects on normal liver cells were observed using 10 mg/mL Lf. Moreover, HepG2 cell apoptosis was significantly increased upon Lf treatment at 10 mg·mL^−1^ [30]. The human MDA‐MB‐231 cell line is widely used as a model in breast cancer research and for studying triple‐negative breast cancer development [53]. MDA‐MB‐231 cell viability was reported to decrease to 80% at 10 mg·mL^−1^ Lf in a study conducted to evaluate the antitumor effects of the exosomal form of bovine milk Lf on MDA‐MB‐231 cells [31].

A study on mesenchymal stem cell secretomes demonstrates that high‐abundance bovine proteins in FBS can mask low‐abundance secreted proteins, suggesting the use of serum‐free conditions for secretome analysis [54, 55]. Moreover, FBS is a natural source of transferrin and as Lf is an 80‐kd non‐heme‐associated iron‐binding glycoprotein of the serum transferrin gene family [56], its absence from FBS cannot be ruled out. The presence of ACh and AChE in FBS, commonly used in cell cultures, is also well‐documented [19, 57, 58] which hinders our ability to assay their secreted levels in our system. To test our hypothesis that ACh and AChE in the cell culture supernatant play a role in regulating Lf function in NSCLC cells, using serum‐free media ensuring careful removal of residual serum components is, therefore, necessary to eliminate the background signal from FBS‐derived ACh and AChE.

**Fig. 2:**
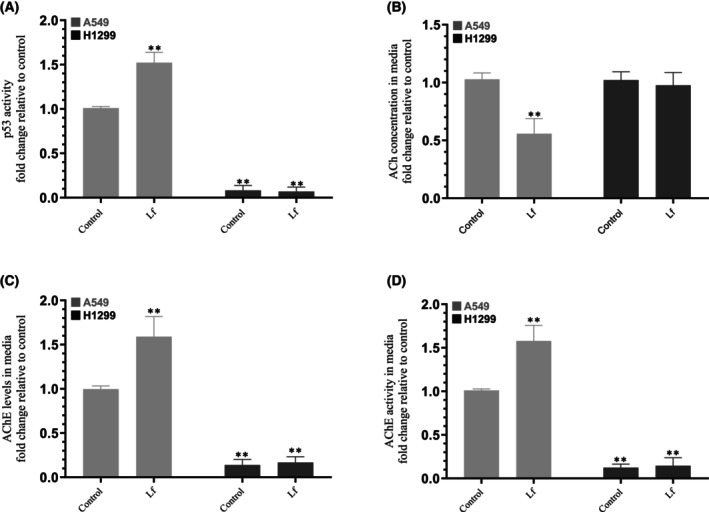
Treatment of A549 cells with lactoferrin (Lf) decreased acetylcholine (ACh) levels and enhanced the activities of p53 and acetylcholinesterase (AChE). Cells (0.2 × 10^5^) were grown in 10% FBS‐supplemented media for 24 h. The following day, the cell monolayers were incubated in serum‐free media for 24 h, then the media were replaced with fresh serum‐free media. The cells were then treated as indicated for 72 h with Lf (10 mg·mL^−1^). The activity of p53 (A) in cell lysates, the levels of ACh (B), the levels of AChE (C), and activity (D) in the media were measured as described in the Materials and methods section. The same amount of protein (3 μL of 600 μg·mL^−1^ total protein) was used for all assays. The graphs summarize the results expressed as means ± SD (*n* = 5) using the graphpad 10.5.0 software. Fold change was calculated relative to the control of each cell line (B) or to the A549 control (A, C, D). Asterisks indicate a statistically significant difference from the corresponding control of each cell line, Mann–Whitney test, **P < 0.01. Absence of asterisks indicates no significance.

The activity of p53 in cell lysates, the levels of ACh and AChE levels and activity in the media (Fig. 2, Table 2) were measured as described in the Materials and methods section. Treatment of A549 cells with Lf led to a ~ 1.50‐fold increase in the activity of p53 (Fig. 2A) while no activity was measured when using H1299 cells which is expected since H1299 cells are known to be p53‐null [1, 33, 59, 60]. The levels of ACh in the media, on the other hand, were found to decrease ~ 1.80‐fold (Fig. 2B) upon A549 cell treatment with Lf while no significant difference was found when using H1299 cell, a result that might be due to the minimal expression of AChE in this cell line as we found previously [13, 17, 39]. This hypothesis was supported by the observation that while no effect was observed when using H1299 cells, Lf stimulated both the levels (Fig. 2C) and activity of AChE (Fig. 2D) ~ 1.60‐fold in A549 cell media.

**Table 2:** Summary of p53 Activity, ACh, and AChE measurements upon Lf treatment. These data show that Lf treatment activates p53 and enhances both AChE levels and activity in A549 cells, while reducing ACh concentration in the media. In contrast, H1299 cells (p53‐null with minimal AChE expression) showed no significant response to Lf under these conditions.

Measurement	A549 cells	H1299 cells
p53 activity	~ 1.50‐fold increase	No activity (p53‐null)
ACh levels in media	~ 1.80‐fold decrease	No significant change
AChE levels in media	~ 1.60‐fold increase	No significant change
AChE activity in media	~ 1.60‐fold increase	No significant change

### The levels of VEGF in the media of A549 and H1299 cells decreased upon treatment with Lf, and in the case of A549 cells, in a manner dependent on p53 and AChE

In this study, we found that treatment of A549 cells with Lf led to activation of p53 and AChE and diminished ACh levels (Fig. 2). These findings might suggest a potential mechanism through which Lf decreases cell viability more effectively in A549 cells as compared to H1299 cells (Fig. 1). Bovine Lf (bLf) was previously reported to decrease proliferation of A549 cells by suppressing the expression of VEGF in a dose‐dependent manner [5, 6, 46]. Tumor growth using a murine model of lung cancer overexpressing VEGF was inhibited by bLf, and A549 cell growth was diminished by bLf *in vitro* [8, 46]. We previously found that nicotine enhances VEGF signaling in NSCLC by positively acting via the α7nAChR and β‐ARs [21]. Our results showed that nicotine treatment led to enhanced levels of VEGF and activities of PI3K, AKT, NFκB, and decreased p53 activity, resulting in increased cell survival and decreased apoptosis [21].

**Fig. 3:**
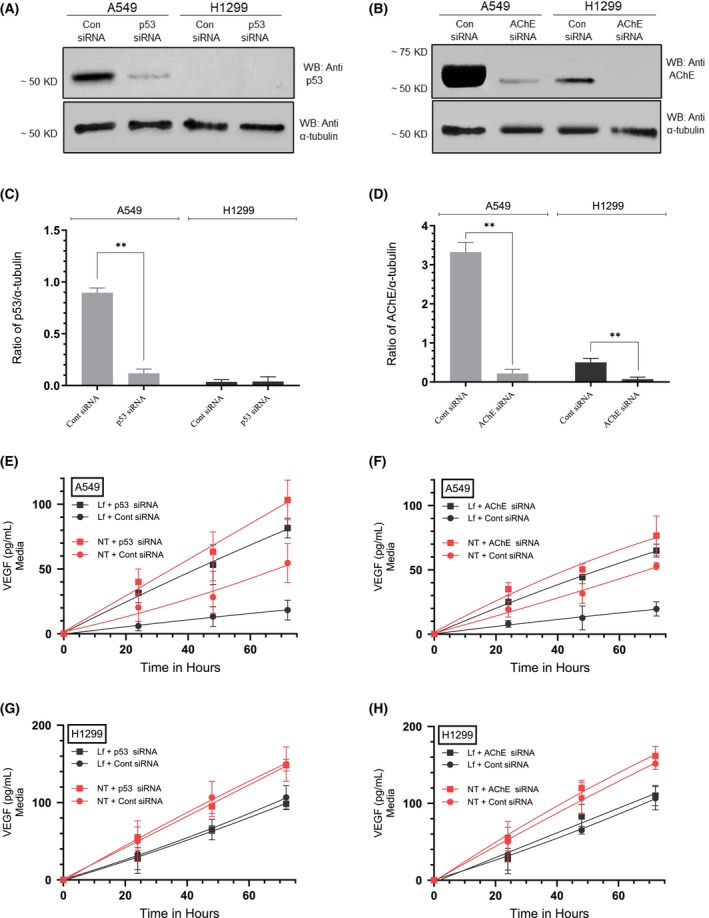
Treatment with lactoferrin (Lf) decreased the levels of vascular endothelial growth factor (VEGF) in the media of A549 and H1299 cells and counteracted the increased levels of VEGF in the media of A549 cells transfected with either p53 or acetylcholinesterase (AChE). Cells (0.2 × 10^5^) were grown in 10% FBS‐supplemented media for 24 h. The following day, the cell monolayers were incubated in serum‐free media for 24 h, then the media was replaced with fresh serum‐free media (0 h). The cells were then incubated with the indicated siRNAs and either not treated (NT) or treated with Lf (10 mg·mL^−1^) as described in the Materials and methods section. The same concentration of total protein (15 μL of 600 μg·mL^−1^) of the cell lysates after transfection for 72 h (A–D) was used for western blotting using the indicated antibodies. As a loading control, anti‐α‐tubulin antibodies were used. The same amount of protein (3 μL of 600 μg·mL^−1^ total protein) of the media was used to quantitate the levels of VEGF as a function of time (E–H) (Materials and methods). Data from five independent assays, each carried out in triplicate, were averaged. The data summarize the results expressed as means ± SD (*n* = 5) using the graphpad 10.5.0 software. Asterisks indicate a statistically significant difference from the corresponding control of each cell line, Mann–Whitney test, **P < 0.01. Absence of asterisks indicates no significance.

To test whether the levels of VEGF are affected by A549 and H1299 cells under our conditions, cells were grown in FBS‐supplemented media for 24 h. The following day, the cell monolayers were incubated in serum‐free media overnight, then the media were replaced with fresh serum‐free media (0 h) (Fig. 3, Table 3). The cells were then incubated with the indicated siRNAs and either not treated or treated with Lf as described in the Materials and methods section.

**Table 3:** Summary of VEGF levels in siRNA‐transfected and Lf‐treated cells.

Condition	Cell line	VEGF fold change	Interpretation
Lf vs. untreated (control siRNA)	A549	~ 3.00‐fold ↓	Lf suppresses VEGF
p53 siRNA vs. control siRNA (untreated)	A549	~ 1.85‐fold ↑	p53 suppresses VEGF
Lf + p53 siRNA vs. p53 siRNA only	A549	~ 1.25‐fold ↓	Lf still reduces VEGF even with p53 knockdown
Lf + p53 siRNA vs. Lf + control siRNA	A549	~ 4.55‐fold ↑	Loss of p53 blocks Lf effect
AChE siRNA vs. control siRNA (untreated)	A549	~ 1.45‐fold ↑	AChE may suppress VEGF
Lf + AChE siRNA vs. AChE siRNA only	A549	~ 1.15‐fold ↓	Lf modestly suppresses VEGF without AChE
Lf + AChE siRNA vs. Lf + control siRNA	A549	~ 3.25‐fold ↑	Loss of AChE blocks Lf effect

H1299 cell summary		
Condition	VEGF fold change	Interpretation
Lf vs. untreated (control siRNA)	~ 1.40‐fold ↓	Lf modestly suppresses VEGF
p53 siRNA vs. control siRNA (untreated)	No effect	p53‐null, no impact
AChE siRNA vs. control siRNA (untreated)	No effect	Minimal AChE expression

The levels of VEGF were reduced ~ 3.00‐fold upon treatment of A549 cells transfected with control siRNA with Lf after 72 h compared to untreated cells (Fig. 3A,C,E). VEGF levels increased ~ 1.85‐fold in A549 cells untreated and transfected with p53 siRNA compared to untreated A549 cells transfected with control siRNA. This finding suggests that p53 is involved in regulating VEGF levels, consistent with our recent report [21]. Since our results (Fig. 2A) show increased p53 activity in A549 cells treated with Lf, we tested the effect of p53 knockdown on the levels of VEGF in A549 cells treated with Lf (Fig. 3A,C,E). In A549 cells transfected with p53 siRNA, Lf treatment decreased the levels of VEGF ~ 1.25‐fold compared to control. Relative to A549 cells transfected with control siRNA and treated with Lf, VEGF levels increased ~ 4.55‐fold under the same conditions when using A549 cells transfected with p53 siRNA (Fig. 3A,C,E). Taken together, these results show that Lf downregulates the levels of VEGF in A549 cells in a manner dependent on p53.

Similar trends but somewhat reduced effects were observed upon AChE knockdown in A549 cells (Fig. 3B,D,F). VEGF levels increased ~ 1.45‐fold in A549 cells untreated and transfected with AChE siRNA compared to untreated A549 cells transfected with control siRNA. This finding suggests that AChE is involved in regulating VEGF levels. Since our results (Fig. 2C,D) show increased AChE levels and activity in A549 cells treated with Lf, we tested the effect of AChE knockdown on the levels of VEGF in A549 cells treated with Lf (Fig. 3B,D,F). In A549 cells transfected with AChE siRNA, Lf treatment for 72 h decreased the levels of VEGF ~ 1.15‐fold compared to control. Relative to A549 cells transfected with control siRNA and treated with Lf, VEGF levels increased ~ 3.25‐fold under the same conditions when using A549 cells transfected with AChE siRNA (Fig. 3B,D,F). Taken together, these results show that Lf downregulates the levels of VEGF in A549 cells in a manner dependent on AChE.

The levels of VEGF were reduced ~ 1.40‐fold upon treatment of H1299 cells transfected with control siRNA with Lf after 72 h compared to untreated cells (Fig. 3A,C,G). No effects were observed using H1299 cells transfected with p53 siRNA (Fig. 3A,C,G) or AChE siRNA (Fig. 3B,D,H) compared to control siRNA‐transfected cells, a finding that is due to the lack of p53 expression in H1299 cells and the minimal levels of AChE in this cell line (Fig. 3B) and as we reported earlier [13, 17, 36, 39].

### Cell treatment with Lf decreased the AKT activity, an effect enhanced by cotreatment using VEGF antibodies in both cell lines and decreased by cotreatment with siRNA targeted against p53 or AChE in A549 cells

Lf was shown to be a potent inducer of apoptosis in a wide range of cancers via mechanisms that include caspase‐3 activation and downregulation of AKT signaling [4, 5, 6, 29]. Earlier reports have shown that VEGF can stimulate AKT activation, driving proliferation, growth, and lung tumor cell survival [61, 62]. Recently, we showed that the addition of anti‐VEGF antibodies to A549 and H1299 cell media inhibited AKT activation [21]. We also reported that blocking casein kinase 2 activity and hyaluronan‐CD44 signaling decreased the activity of AKT and led to enhanced p53 activation in A549 cells [38] and that the treatment of A549 cells with the p53 inhibitor, pifithrin‐α, led to upregulation of the activity of AKT [23, 40]. Whether AChE is involved in Lf‐dependent regulation of AKT activity in NSCLC cells is not known. In this study, we examined the effect of Lf on the activity of AKT in A549 and H1299 cells and the possible involvement of p53, AChE, and VEGF in the process.

**Fig. 4:**
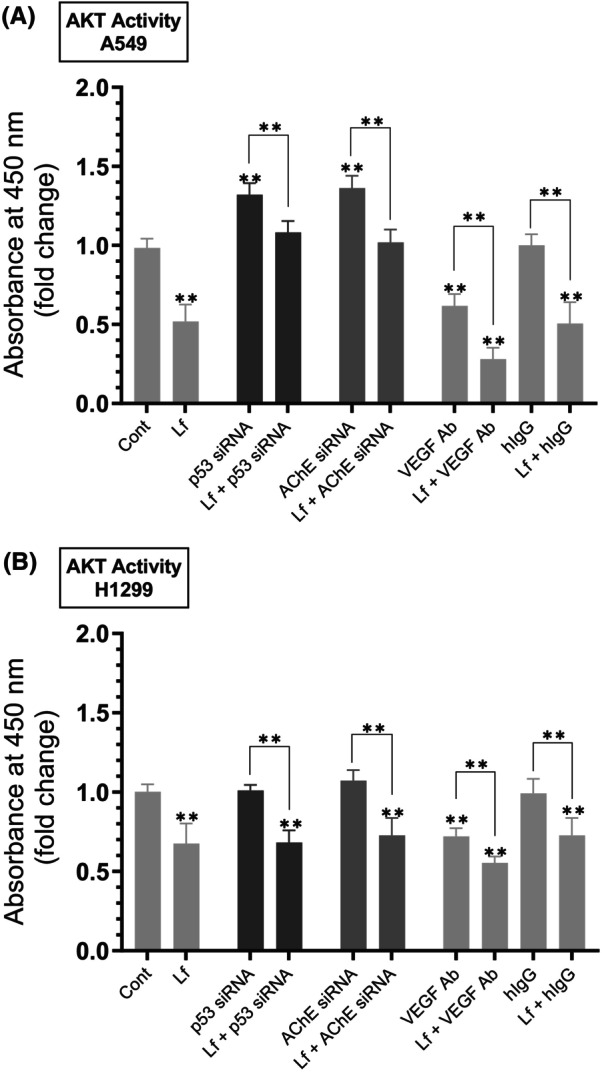
The AKT activity decreased by cell treatment with lactoferrin (Lf), an effect diminished by cotreatment with siRNA targeted against p53 or acetylcholinesterase (AChE) in A549 cells but enhanced by cotreatment using vascular endothelial growth factor (VEGF) antibodies (Ab) in both cell lines. Cells (0.2 × 10^5^) were grown in 10% FBS‐supplemented media for 24 h. The following day, the cell monolayers were serum starved for 24 h, then incubated in serum‐free media for 72 h in the absence or presence of Lf (10 mg·mL^−1^), control siRNA (100 nm), p53 siRNA (100 nm), AChE siRNA (100 nm), hIgG (20 μg·mL^−1^) as a control, anti‐VEGF‐specific antibodies (20 μg·mL^−1^), or in combination. The AKT activity (A, B) was measured as described in the Materials and methods section. Data from five independent assays, each carried out in triplicate, were averaged, normalized, and expressed as fold change relative to control untreated cells (Cont) using the graphpad 10.5.0 software. The data summarize the results expressed as means ± SD (*n* = 5). Asterisks indicate a statistically significant difference from the corresponding control of each cell line, Mann–Whitney test. Absence of asterisks indicates no significance. Statistical differences between different groups were analyzed by an ordinary one‐way analysis of variance (ANOVA) followed by Tukey's *post hoc* multiple comparison test, **P < 0.01.

Cells were grown in FBS‐supplemented media for 24 h, serum‐starved overnight, then incubated in serum‐free media for 72 h in the absence or presence of Lf, control siRNA, p53 siRNA, AChE siRNA, hIgG as a control, anti‐VEGF‐specific antibodies, or in combination. The AKT activity (Fig. 4, Table 4) was measured as described in the Materials and methods section. Treatment of A549 cells with Lf decreased the activity of AKT ~ 1.95‐fold (Fig. 4A) and ~ 1.50‐fold in H1299 cells (Fig. 4B). A549 cell incubation with p53 siRNA led to ~ 1.35‐fold activation of AKT. Relative to treatment using only p53 siRNA, co‐incubation of A549 cells with p53 siRNA and Lf resulted in ~ 1.22‐fold decrease in the activity of AKT, a reduction smaller than that observed in the absence (~ 1.95‐fold) of p53 siRNA (Fig. 4A). This finding might suggest that p53 is an important regulator of the Lf‐dependent inhibition of AKT activity in A549 cells. Knockdown of AChE in A549 cells increased the AKT activity ~ 1.35‐fold. Relative to treatment with AChE siRNA, cotreatment of A549 cells with AChE siRNA and Lf decreased the AKT activity ~ 1.35‐fold, an effect diminished compared to cell treatment in the absence of AChE siRNA (~ 1.95‐fold), suggesting that AChE might also play a role in the Lf‐dependent inhibition of AKT in A549 cells (Fig. 4A). The AKT activities measured in H1299 cells treated with either p53‐ or AChE siRNA (Fig. 4B) were similar to those obtained using control cells, a finding likely due to the lack of p53 in this cell line (Fig. 3A) and the minimal expression of AChE (Fig. 3B) compared to A549 cells. To test the effect of VEGF on the activity of AKT upon cell treatment with Lf, we neutralized the function of VEGF using a VEGF neutralizing antibody previously used in NSCLC cells [62]. Earlier, we reported that addition of this anti‐VEGF antibody to A549 and H1299 cells decreased cell viability, increased apoptosis, blocked AKT function, and increased p53 activation in A549 cells [21]. Compared to the hIgG control, treatment of A549 cells with VEGF antibodies resulted in ~ 1.60‐fold decrease in the activity of AKT (Fig. 4A). Compared to treatment with only the VEGF antibody, cotreatment of A549 cells with both the VEGF antibodies and Lf resulted in ~ 2.20‐fold decrease in the activity of AKT, suggesting that cell cotreatment is more effective at inhibiting AKT than each treatment alone (Fig. 4A). Similarly, compared to hIgG control, treatment of H1299 cells with VEGF antibodies resulted in ~ 1.35‐fold decrease in the activity of AKT (Fig. 4B). Cotreatment of H1299 cells with both the VEGF antibodies and Lf resulted in ~ 1.30‐fold decrease in the activity of AKT relative to treatment with only the VEGF antibody suggesting that, as observed in A549 cells, cotreatment is more effective at inhibiting AKT than each treatment alone (Fig. 4B).

**Table 4:** Summary of AKT activity under various treatments.

Condition	Cell line	AKT activity fold change
Lf treatment	A549	~ 1.95‐fold ↓
p53 siRNA	A549	~ 1.35‐fold ↑
Lf + p53 siRNA vs. p53 siRNA	A549	~ 1.22‐fold ↓
AChE siRNA	A549	~ 1.35‐fold ↑
Lf + AChE siRNA vs. AChE siRNA	A549	~ 1.35‐fold ↓
VEGF antibody	A549	~ 1.60‐fold ↓
VEGF antibody + Lf vs. VEGF antibody	A549	~ 2.20‐fold ↓

H1299 cell summary	
Condition	AKT activity fold change
Lf treatment	~ 1.50‐fold ↓
p53 or AChE siRNA	No significant change
VEGF antibody	~ 1.35‐fold ↓
VEGF antibody + Lf vs. VEGF antibody	~ 1.30‐fold ↓

### Lf‐induced activation of caspase‐3 was reduced by A549 cell cotreatment with siRNA targeted against p53 and/or AChE and, conversely, augmented by blocking the function of VEGF and/or AKT in both cell lines

**Fig. 5:**
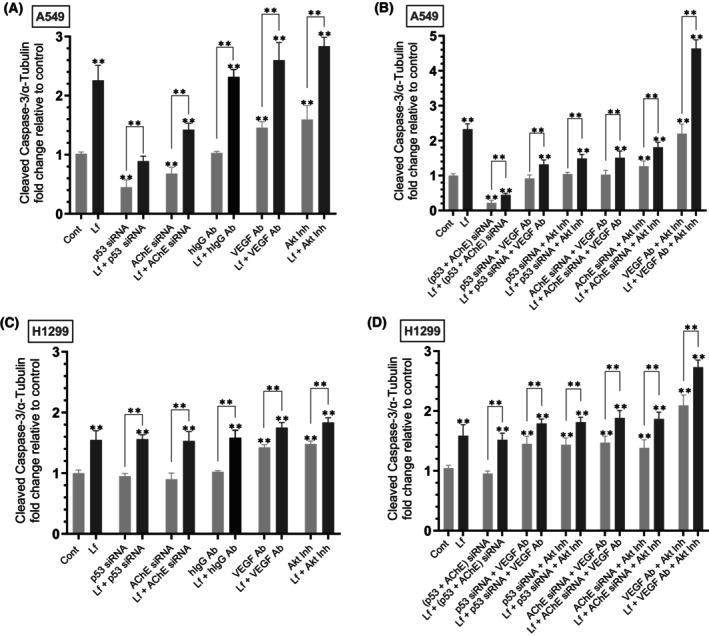
Cleaved caspase‐3 activity increased by lactoferrin (Lf) treatment is antagonized by cotreatment with siRNA targeted against p53 and/or acetylcholinesterase (AChE) in A549 cells and, conversely, augmented by inhibiting vascular endothelial growth factor (VEGF) and/or AKT function in both cell lines. Cells (0.2 × 10^5^) were grown in 10% FBS‐supplemented media for 24 h. The following day, the cell monolayers were serum starved for 24 h, then incubated in serum‐free media for 72 h in the absence or presence of Lf (10 mg·mL^−1^), control siRNA (100 nm), p53 siRNA (100 nm), AChE siRNA (100 nm), hIgG (20 μg·mL^−1^) as a control, anti‐VEGF‐specific antibodies (20 μg·mL^−1^), AKT inhibitor (1.75 μm), or in combination. Cleaved caspase‐3 activity (A–D) was measured as described in the Materials and methods section. Data from five independent assays, each carried out in triplicate, were averaged, normalized, and expressed as fold change relative to control untreated cells (Cont) using the graphpad 10.5.0 software. The data summarize the results expressed as means ± SD (*n* = 5). Asterisks indicate a statistically significant difference from the control of each cell line, Mann–Whitney test. Statistical differences between different groups were analyzed by an ordinary one‐way analysis of variance (ANOVA) followed by Tukey's *post hoc* multiple comparison test, **P < 0.01.

To examine whether Lf affects the activity of caspase‐3, cells were grown in FBS‐supplemented media for 24 h. The following day, the cell monolayers were serum‐starved overnight, then incubated in serum‐free media for 72 h in the absence or presence of Lf, control siRNA, p53 siRNA, AChE siRNA, hIgG as a control, anti‐VEGF‐specific antibodies, AKT inhibitor, or in combination. Caspase‐3 activity (Fig. 5, Table 5) was measured as described in the Materials and methods section.

**Table 5:** Summary of cleaved caspase‐3 activity under various treatments.

A549 cell summary	
Condition	Fold change
Lf treatment	~ 2.25‐fold ↑
p53 siRNA	~ 2.20‐fold ↓
Lf + p53 siRNA vs. p53 siRNA	~ 1.95‐fold ↑
AChE siRNA	~ 1.45‐fold ↓
Lf + AChE siRNA vs. AChE siRNA	~ 2.00‐fold ↑
VEGF antibody	~ 1.45‐fold ↑
Lf + VEGF antibody vs. VEGF antibody	~ 1.75‐fold ↑
AKT inhibitor	~ 1.60‐fold ↑
Lf + AKT inhibitor vs. AKT inhibitor	~ 1.75‐fold ↑
p53 + AChE siRNA	~ 4.60‐fold ↓
Lf + p53 + AChE siRNA vs. p53 + AChE siRNA	~ 2.00‐fold ↑
Lf + p53 siRNA + VEGF antibody vs. same w/o Lf	~ 1.40‐fold ↑
Lf + p53 siRNA + AKT inhibitor vs. same w/o Lf	~ 1.40‐fold ↑
Lf + AChE siRNA + VEGF antibody vs. same w/o Lf	~ 1.50‐fold ↑
Lf + AChE siRNA + AKT inhibitor vs. same w/o Lf	~ 1.40‐fold ↑
VEGF antibody + AKT inhibitor	~ 2.20‐fold ↑
Lf + VEGF antibody + AKT inhibitor vs. same w/o Lf	~ 2.15‐fold ↑

H1299 cell summary	
Condition	Fold change
Lf treatment	~ 1.55‐fold ↑
p53 or AChE siRNA	No effect
VEGF antibody	~ 1.45‐fold ↑
Lf + VEGF antibody vs. VEGF antibody	~ 1.22‐fold ↑
AKT inhibitor	~ 1.45‐fold ↑
Lf + AKT inhibitor vs. AKT inhibitor	~ 1.25‐fold ↑
p53 + AChE siRNA ± Lf	No effect
Lf + p53/AChE siRNA + VEGF antibody	No difference vs. Lf + VEGF antibody
Lf + p53/AChE siRNA + AKT inhibitor	No difference vs. Lf + AKT inhibitor
VEGF antibody + AKT inhibitor	~ 2.00‐fold ↑
Lf + VEGF antibody + AKT inhibitor vs. same w/o Lf	~ 1.30‐fold ↑

Treatment of A549 cells with Lf resulted in a ~ 2.25‐fold increase in cleaved caspase‐3 (Fig. 5A). Incubation of A549 cells with p53 siRNA decreased cleaved caspase‐3 ~ 2.20‐fold relative to control, a result consistent with the role p53 plays in apoptosis. Cotreatment with Lf + p53 siRNA increased cleaved caspase‐3 ~ 1.95‐fold compared to A549 cells transfected with p53 siRNA, an effect lower than that observed by A549 cell treatment with only Lf (~ 2.25‐fold relative to control) suggesting a role of p53 in Lf‐induced activation of caspase‐3 in A549 cells. A549 cells treated with AChE siRNA decreased cleaved caspase‐3 ~ 1.45‐fold relative to control, a finding suggesting a role of AChE in promoting apoptosis in this cell line. Relative to A549 cells treated with only AChE siRNA, cotreatment with Lf + AChE siRNA increased cleaved caspase‐3 ~ 2.00‐fold, an effect lower than that observed by A549 cell treatment with only Lf (~ 2.25‐fold) suggesting a role of AChE in Lf‐induced activation of caspase‐3 in A549 cells. A549 cells incubated with VEGF antibodies led to a ~ 1.45‐fold increase in cleaved caspase‐3 relative to control, while cotreatment with Lf + VEGF antibodies resulted in a ~ 1.75‐fold increase compared to treatment with only VEGF antibodies. A549 cells incubated with the AKT inhibitor led to a ~ 1.60‐fold increase in cleaved caspase‐3 relative to control, while cotreatment with Lf + the AKT inhibitor resulted in a ~ 1.75‐fold increase in cleaved caspase‐3 relative to A549 cell treatment with only the AKT inhibitor.

Cotreatment of A549 cells with (p53 + AChE) siRNA (Fig. 5B) decreased cleaved caspase‐3 ~ 4.60‐fold, an effect greater than that observed by treatment with only p53 siRNA or AChE siRNA (Fig. 5A). This effect was, in part, reversed by co‐incubation of A549 cells with Lf + (p53 + AChE) siRNA (~ 2.00‐fold increase in cleaved caspase‐3) compared to treatment without Lf. Cotreatment of A549 cells with Lf + p53 siRNA + the VEGF antibody (Fig. 5B) increased cleaved caspase‐3 ~ 1.40‐fold compared to the same treatment but without Lf. Cotreatment of A549 cells with Lf + p53 siRNA + the AKT inhibitor also increased cleaved caspase‐3 ~ 1.40‐fold compared to the same treatment but without Lf. Similarly, relative to cells treated under the same conditions but without Lf, cleaved caspase‐3 increased as follows: (Lf + AChE siRNA + VEGF antibody ~ 1.50‐fold increase), (Lf + AChE siRNA + the AKT inhibitor ~ 1.40‐fold increase). Relative to control, cleaved caspase‐3 increased ~ 2.20‐fold upon A549 cell incubation with the VEGF antibody and the AKT inhibitor. Compared to the same treatment but without Lf, cleaved caspase‐3 increased ~ 2.15‐fold upon incubation with Lf + the VEGF antibody + the AKT inhibitor. Collectively, these results seem to suggest that Lf induces activation of caspase‐3 in A549 cells in a manner involving p53, AChE, VEGF, and AKT.

Treatment of H1299 cells with Lf resulted in a ~ 1.55‐fold increase in cleaved caspase‐3 (Fig. 5C). No differences were found upon incubation of H1299 cells with p53 siRNA or AChE siRNA which is consistent with the lack of p53 (Fig. 3A) and minimal expression of AChE (Fig. 3B) in this cell line and with previous reports [13, 33, 39, 60]. H1299 cells incubated with VEGF antibodies led to a ~ 1.45‐fold increase in cleaved caspase‐3 relative to control, while cotreatment with Lf + VEGF antibodies resulted in a ~ 1.22‐fold increase compared to treatment with only VEGF antibodies (Fig. 5C). H1299 cells incubated with the AKT inhibitor led to a ~ 1.45‐fold increase in cleaved caspase‐3 relative to control, while cotreatment with Lf + the AKT inhibitor resulted in a ~ 1.25‐fold increase in cleaved caspase‐3 compared to treatment with only the AKT inhibitor (Fig. 5C).

Cotreatment of H1299 cells with (p53 + AChE) siRNA (Fig. 5D) in the absence or presence of Lf had no effect on cleaved caspase‐3 compared to the corresponding controls. Results obtained by cotreatment of H1299 cells with Lf + p53‐ or AChE siRNA + the VEGF antibody, or Lf + p53‐ or AChE siRNA + the AKT inhibitor (Fig. 5D) were indistinguishable from the same treatment but in the absence of p53 or AChE siRNAs (Fig. 5C). Relative to control, cleaved caspase‐3 increased ~ 2.00‐fold upon H1299 cell incubation with the VEGF antibody and the AKT inhibitor (Fig. 5D). Compared to the same treatment but without Lf, cleaved caspase‐3 increased ~ 1.30‐fold upon incubation with Lf + the VEGF antibody + the AKT inhibitor. Collectively, these results seem to suggest that Lf induces activation of caspase‐3 in H1299 cells in a manner involving VEGF and AKT.

### The effect of Lf treatment on cell viability is reduced by cotreatment with siRNA targeted against p53 and/or AChE in A549 cells and, conversely, augmented by blocking the function of VEGF and/or AKT in both cell lines

**Fig. 6:**
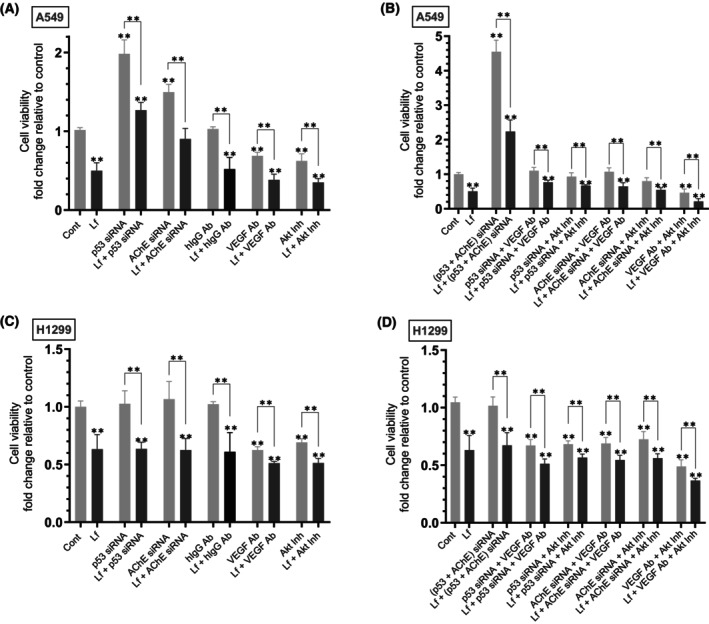
The decrease in cell viability by lactoferrin (Lf) treatment is antagonized by cotreatment with siRNA targeted against p53 and/or acetylcholinesterase (AChE) in A549 cells and, conversely, augmented by inhibiting vascular endothelial growth factor (VEGF) and/or AKT function in both cell lines. Cells (0.2 × 10^5^) were grown in 10% FBS‐supplemented media for 24 h. The following day, the cell monolayers were serum‐starved for 24 h, then incubated in serum‐free media for 72 h in the absence or presence of Lf (10 mg·mL^−1^), control siRNA (100 nm), p53 siRNA (100 nm), AChE siRNA (100 nm), hIgG (20 μg·mL^−1^) as a control, anti‐VEGF‐specific antibodies (20 μg·mL^−1^), AKT inhibitor (1.75 μm), or in combination. Cell viability (A–D) was measured as described in the Materials and methods section. Data from five independent assays, each carried out in triplicate, were averaged, normalized, and expressed as fold change relative to control untreated cells (Cont) using the graphpad 10.5.0 software. The data summarize the results expressed as means ± SD (*n* = 5). Asterisks indicate a statistically significant difference from the control of each cell line, Mann–Whitney test. Statistical differences between different groups were analyzed by an ordinary one‐way analysis of variance (ANOVA) followed by Tukey's *post hoc* multiple comparison test, **P < 0.01.

To examine the effect of Lf on cell viability, cells were grown in FBS‐supplemented media overnight, then serum starved for 24 h. The cells were then incubated in serum‐free media for 72 h in the absence or presence of Lf, p53 siRNA, AChE siRNA, hIgG control, anti‐VEGF‐specific antibodies, AKT inhibitor, or in combination. Cell viability (Fig. 6, Table 6) was measured as described in the Materials and methods section.

**Table 6:** Summary of cell viability under various treatments.

A549 cell summary	
Condition	Fold change
Lf treatment	~ 2.00‐fold ↓
p53 siRNA	~ 2.00‐fold ↑
Lf + p53 siRNA vs. p53 siRNA	~ 1.55‐fold ↓
AChE siRNA	~ 1.50‐fold ↑
Lf + AChE siRNA vs. AChE siRNA	~ 1.65‐fold ↓
VEGF antibody	~ 1.45‐fold ↓
Lf + VEGF antibody vs. VEGF antibody	~ 1.75‐fold ↓
AKT inhibitor	~ 1.60‐fold ↓
Lf + AKT inhibitor vs. AKT inhibitor	~ 1.75‐fold ↓
p53 + AChE siRNA	~ 4.55‐fold ↑
Lf + p53 + AChE siRNA vs. p53 + AChE siRNA	~ 2.00‐fold ↓
Lf + p53 siRNA + VEGF antibody vs. same w/o Lf	~ 1.45‐fold ↓
Lf + p53 siRNA + AKT inhibitor vs. same w/o Lf	~ 1.40‐fold ↓
Lf + AChE siRNA + VEGF antibody vs. same w/o Lf	~ 1.65‐fold ↓
Lf + AChE siRNA + AKT inhibitor vs. same w/o Lf	~ 1.45‐fold ↓
VEGF antibody + AKT inhibitor	~ 2.15‐fold ↓
Lf + VEGF antibody + AKT inhibitor vs. same w/o Lf	~ 2.15‐fold ↓

H1299 cell summary	
Condition	Fold change
Lf treatment	~ 1.60‐fold ↓
p53 or AChE siRNA	No effect
VEGF antibody	~ 1.60‐fold ↓
Lf + VEGF antibody vs. VEGF antibody	~ 1.20‐fold ↓
AKT inhibitor	~ 1.45‐fold ↓
Lf + AKT inhibitor vs. AKT inhibitor	~ 1.35‐fold ↓
p53 + AChE siRNA ± Lf	No effect
Lf + p53/AChE siRNA + VEGF antibody	No difference vs. Lf + VEGF antibody
Lf + p53/AChE siRNA + AKT inhibitor	No difference vs. Lf + AKT inhibitor
VEGF antibody + AKT inhibitor	~ 2.00‐fold ↓
Lf + VEGF antibody + AKT inhibitor vs. same w/o Lf	~ 1.35‐fold ↓

Treatment of A549 cells with Lf resulted in ~ 2.00‐fold decrease in cell viability (Fig. 6A). Incubation of A549 cells with p53 siRNA increased viability ~ 2.00‐fold relative to control. Relative to p53 siRNA treatment alone, cotreatment with Lf + p53 siRNA decreased viability ~ 1.55‐fold, an effect lower than that observed by A549 cell treatment with only Lf (~ 2.00‐fold) suggesting that Lf decreases A549 cell viability in a manner involving p53. A549 cells treated with AChE siRNA increased viability ~ 1.50‐fold relative to control, a finding suggesting a role of AChE in decreasing cell viability. Relative to A549 cells treated with only AChE siRNA, cotreatment with Lf + AChE siRNA decreased cell viability ~ 1.65‐fold, an effect lower than that observed by A549 cell treatment with only Lf (~ 2.00‐fold) suggesting that Lf decreases A549 cell viability in a manner involving AChE. A549 cells incubated with VEGF antibodies led to ~ 1.45‐fold decrease in viability relative to hIgG control, while cotreatment with Lf + VEGF antibodies resulted in ~ 1.75‐fold decrease compared to treatment with only VEGF antibodies. A549 cells incubated with the AKT inhibitor led to ~ 1.60‐fold decrease in viability relative to control, while cotreatment with Lf + the AKT inhibitor resulted in ~ 1.75‐fold decrease in cell viability compared to treatment using only the AKT inhibitor.

Cotreatment of A549 cells with (p53 + AChE) siRNA (Fig. 6B) increased viability ~ 4.55‐fold, an effect greater than that observed by treatment with only p53 siRNA or AChE siRNA (Fig. 6A). This effect was, in part, reversed by co‐incubation of A549 cells with Lf + (p53 + AChE) siRNA (~ 2.00‐fold decrease in cell viability) compared to treatment without Lf. Cotreatment of A549 cells with Lf + p53 siRNA + the VEGF antibody (Fig. 6B) decreased cell viability ~ 1.45‐fold compared to the same treatment but without Lf. Cotreatment of A549 cells with Lf + p53 siRNA + the AKT inhibitor also decreased cell viability ~ 1.40‐fold compared to the same treatment but without Lf. Similarly, relative to cells treated under the same conditions but without Lf, cell viability decreased as follows: (Lf + AChE siRNA + VEGF antibody ~ 1.65‐fold decrease), (Lf + AChE siRNA + the AKT inhibitor ~ 1.45‐fold decrease). Relative to control, cell viability decreased ~ 2.15‐fold upon A549 cell incubation with the VEGF antibody and the AKT inhibitor. Compared to the same treatment but without Lf, cell viability decreased ~ 2.15‐fold upon incubation with Lf + the VEGF antibody + the AKT inhibitor. Collectively, these results seem to suggest that Lf decreases cell viability in A549 cells in a manner involving p53, AChE, VEGF, and AKT.

Treatment of H1299 cells with Lf resulted in ~ 1.60‐fold decrease in cell viability (Fig. 6C). No differences were found upon incubation of H1299 cells with p53 siRNA or AChE siRNA which is consistent with the lack of p53 (Fig. 3A,C) and minimal expression of AChE (Fig. 3B,D) in this cell line and with previous reports [13, 33, 39, 60]. H1299 cells incubated with VEGF antibodies led to ~ 1.60‐fold decrease in cell viability relative to control, while cotreatment with Lf + VEGF antibodies resulted in ~ 1.20‐fold decrease compared to treatment with only VEGF antibodies. H1299 cells incubated with the AKT inhibitor led to ~ 1.45‐fold decrease in cell viability relative to control, while co‐treatment with Lf + the AKT inhibitor resulted in ~ 1.35‐fold decrease in cell viability compared to treatment with only the AKT inhibitor.

Co‐treatment of H1299 cells with (p53 + AChE) siRNA (Fig. 6D) in the absence or presence of Lf had no effect compared to the corresponding controls. Results obtained by cotreatment of H1299 cells with Lf + p53‐ or AChE siRNA + the VEGF antibody, or Lf + p53‐ or AChE siRNA + the AKT inhibitor (Fig. 6D) were indistinguishable from the same treatment but in the absence of p53 or AChE siRNAs (Fig. 6C). Relative to control, cell viability decreased ~ 2.00‐fold upon H1299 cell incubation with the VEGF antibody and the AKT inhibitor (Fig. 6D). Compared to the same treatment but without Lf, cell viability decreased ~ 1.35‐fold upon incubation with Lf + the VEGF antibody + the AKT inhibitor. Collectively, these results seem to suggest that Lf decreases cell viability in H1299 cells in a manner involving VEGF and AKT.

## Conclusions

Novel results from this study include the findings that (1) treatment with Lf was more effective at decreasing A549 cell viability and activating caspase‐3 compared to H1299 cells. (2) In A549 cells: (a) Lf increased cleaved caspase‐3 by activating p53 and AChE leading to decreased ACh concentrations; (b) AChE plays a role in Lf‐dependent regulation of VEGF levels and AKT activity; and (c) Lf acts to decrease the levels of ACh which ultimately leads to activation of caspase‐3 and decreased cell survival. (3) The effect of Lf on activation of caspase‐3 and cell viability is modulated by knockdown of p53 and/or AChE in A549 cells and by inhibiting VEGF and/or AKT function in both cell lines.

The findings from this study suggest that the Lf‐AChE‐ACh pathway may play a role in regulating cancer cell survival in NSCLC under specific *in vitro* conditions. These observations provide initial insights into the potential involvement of this pathway in tumor biology. However, several limitations must be considered when interpreting these results. The experimental conditions used in this study, including cell lines, serum‐free media, and defined treatment regimens, may not fully replicate the complex tumor microenvironment or the heterogeneity found in patient tumors. Moreover, the expression levels and functional relevance of AChE and related signaling molecules *in vivo* remain to be thoroughly characterized. As such, caution is warranted when extrapolating these findings to clinical contexts. Additional research using animal models and patient‐derived samples will be necessary to validate the pathway's functional significance and therapeutic relevance. Despite these limitations, the Lf‐AChE‐ACh axis remains a potentially interesting target for future translational studies aimed at improving NSCLC treatment strategies.

## Conflict of interest

All authors read and approved the final manuscript and the authors declare no conflict of interest.

## Author contributions

HGE conceived, designed, coordinated the study, supervised the project, and wrote the paper. SG, CW, AS, and BH performed ELISAs, p53‐ and kinase assays, cell viability and apoptosis assays, and transfections. BL, AC, and SS helped with western blotting, transfections, cell viability and apoptosis assays, and ELISAs. JG maintained the cells and provided advice on tissue culture. DH critiqued the manuscript. All authors have read and agreed to the published version of the manuscript.

## Data Availability

The data presented in this study are available in this article.
